# Meningeal Mast Cells Contribute to ATP-Induced Nociceptive Firing in Trigeminal Nerve Terminals: Direct and Indirect Purinergic Mechanisms Triggering Migraine Pain

**DOI:** 10.3389/fncel.2019.00195

**Published:** 2019-05-10

**Authors:** Ksenia Koroleva, Oleg Gafurov, Valeriia Guselnikova, Dilyara Nurkhametova, Raisa Giniatullina, Guzel Sitdikova, Olli S. Mattila, Perttu J. Lindsberg, Tarja Maarit Malm, Rashid Giniatullin

**Affiliations:** ^1^Laboratory of Neurobiology, Kazan Federal University, Kazan, Russia; ^2^A. I. Virtanen Institute for Molecular Sciences, University of Eastern Finland, Kuopio, Finland; ^3^Department of General and Special Morphology, Institute of Experimental Medicine, Saint Petersburg, Russia; ^4^Department of Neurology and Clinical Neurosciences, University of Helsinki and Helsinki University Hospital, Helsinki, Finland

**Keywords:** ATP, 5-HT3, mast cells, pain, migraine

## Abstract

Peripheral mechanisms of primary headaches such as a migraine remain unclear. Meningeal afferents surrounded by multiple mast cells have been suggested as a major source of migraine pain. Extracellular ATP released during migraine attacks is a likely candidate for activating meningeal afferents via neuronal P2X receptors. Recently, we showed that ATP also increased degranulation of resident meningeal mast cells (Nurkhametova et al., 2019). However, the contribution of ATP-induced mast cell degranulation in aggravating the migraine pain remains unknown. Here we explored the role of meningeal mast cells in the pro-nociceptive effects of extracellular ATP. The impact of mast cells on ATP mediated activation of peripheral branches of trigeminal nerves was measured electrophysiologically in the dura mater of adult wild type (WT) or mast cell deficient mice. We found that a spontaneous spiking activity in the meningeal afferents, at baseline level, did not differ in two groups. However, in WT mice, meningeal application of ATP dramatically (24.6-fold) increased nociceptive firing, peaking at frequencies around 10 Hz. In contrast, in mast cell deficient animals, ATP-induced excitation was significantly weaker (3.5-fold). Application of serotonin to meninges in WT induced strong spiking. Moreover, in WT mice, the 5-HT3 antagonist MDL-7222 inhibited not only serotonin but also the ATP induced nociceptive firing. Our data suggest that extracellular ATP activates nociceptive firing in meningeal trigeminal afferents via amplified degranulation of resident mast cells in addition to direct excitatory action on the nerve terminals. This highlights the importance of mast cell degranulation via extracellular ATP, in aggravating the migraine pain.

## Introduction

Mast cells are immune cells implicated in various inflammatory diseases. Since several original studies by Theoharides et al. (1995, 2005), the role of meningeal mast cells as triggers of migraine attacks was further explored by others, showing the pro-nociceptive role of mast cell derived pro-inflammatory cytokines/chemokines (Reuter et al., 2001; Levy et al., 2007; Baun et al., 2012; Conti et al., 2019). We recently showed that serotonin appeared to be the most important neurotransmitter released by degranulated dural mast cells to activate peripheral meningeal nerve fibers (Kilinc et al., 2017). Despite several potential candidates, it remains, however, unclear which signal or chemical agent initially triggers the activation of meningeal mast cells.

In the frame of the current Research Topic, we published a recent study showing that extracellular ATP acts through the P2X7 subtype of purinergic receptors on meningeal mast cells, leading to both mast cell activation and degranulation (Nurkhametova et al., 2019). Similar results were found also in human mast cells line (Wareham and Seward, 2016). Based on these findings, we hypothesized that this mast-cell based mechanism can indirectly contribute to ATP-induced activation of meningeal afferents. Notably, it is well established that ATP directly excites trigeminal nerve terminals (Zhao and Levy, 2015; Yegutkin et al., 2016; Zakharov et al., 2016), mainly via P2X3 receptors (Yegutkin et al., 2016). Thus, ATP potentially may have a dual complementary migraine pain promoting effect. Given a plethora of pro-inflammatory and pro-nociceptive substances released from active mast cells (Conti et al., 2019) these data suggest that ATP-driven mechanisms might significantly contribute both to meningeal neuroinflammation and to prolonged pain in migraine.

Here, we set out to differentiate the indirect, mast cell-mediated, and direct actions of ATP on meningeal afferents in isolated mouse hemiskull preparations, in mice deficient of mast cells. Our data highlight the importance of ATP driven mast cell degranulation in the aggravation of nociceptive firing in migraine pain.

## Materials and Methods

### Animals

Experiments were performed on 10–12-week-old male WT C57BL/6J and C57BL/6J-KitW-v/J mice provided by the Animal Facilities of the University of Eastern Finland (UEF). All procedures were approved by the Committee for the Welfare of Laboratory Animals of the University of Eastern Finland and the Provincial Government of Kuopio. Experiments were conducted according to the European Community Council guidelines (Directives 86/609/EEC). All efforts were made to minimize the number of animals used and their suffering.

### Toluidine Blue Staining of Meningeal Mast Cells

Toluidine Blue staining was used to identify mast cells in meningeal tissues as previously described by Shelukhina et al. (2017) and Nurkhametova et al. (2019). In short, the brains were carefully removed from the hemiskulls leaving the meninges intact on bone tissue. The hemiskulls were filled with artificial cerebrospinal fluid (ACSF) (in mM): NaCl 115, KCl 3, CaCl2 2, MgCl2 1, NaH2PO4 1, NaHCO3 25, glucose 11) for 10 min (room temperature) and oxygenated with 95% O2/ 5% CO2. The hemiskulls were then transferred to 4% paraformaldehyde and fixed overnight at 4°C followed by three washes with phosphate buffered saline (PBS). Meningeal tissues were dissected from hemiskulls and placed on glass slides (Polysine^®^ Thermo-Scientific, United States) for staining with Toluidine Blue (Levy et al., 2007; Kilinc et al., 2017). Images were acquired with an Olympus AX-TFSM microscope (Olympus, Japan).

### Electrophysiology

Isolated whole-mount mouse hemiskulls were used for spike recordings as previously described (Zakharov et al., 2015; Kilinc et al., 2017; Mikhailov et al., 2019). In short, hemiskulls were cleaned from cranial muscles, keeping the dura mater with meningeal nerves and vessels intact. The main meningeal branch of the trigeminal nerve was cleaned from surrounding tissue, cut and placed inside the glass electrode filled with the ACSF. All recordings of electrical activity from trigeminal nerves were performed from hemiskull preparations continuously perfused by ACSF oxygenated with 95% O2/ 5% CO2. Trigeminal nerve spiking activity was registered using DAM80 amplifier (World Precision Instruments, Sarasota, FL, United States). Electrical signals were digitized using a NI PCI6221 board (National Instruments, United States) stored on a PC for off-line analysis. Signals were visualized by WinEDR v.3.2.7 software (University of Strathclyde, Glasgow, United Kingdom) and analyzed with Matlab-based software (Zakharov et al., 2015). All agonists and the antagonist of 5-HT3 receptors (ATP from Sigma-Aldrich, Germany and serotonin and MDL-7222 from Tocris Bioscience, United Kingdom) were prepared immediately before usage and were applied to the receptive fields in meninges by fast perfusion (7 ml/min). ATP and serotonin were dissolved in water, while MDL-7222 was first dissolved in DMSO (stock concentration 30 mM) and then diluted to a final concentration of 10 μM in the basic solution.

### Statistical Analysis

Experimental data were analyzed using Matlab (MathWorks, Inc., United States). Data are presented as mean ± SEM (standard error of mean). The data were analyzed using Student’s paired *t*-test and Mann–Whitney *U*-test when appropriate, the differences accepted significant at *p* ≤ 0.05.

## Results

### ATP Induced Activation of Meningeal Afferents Reduced in Mast Cells Deficient Mice

**FIGURE 1:**
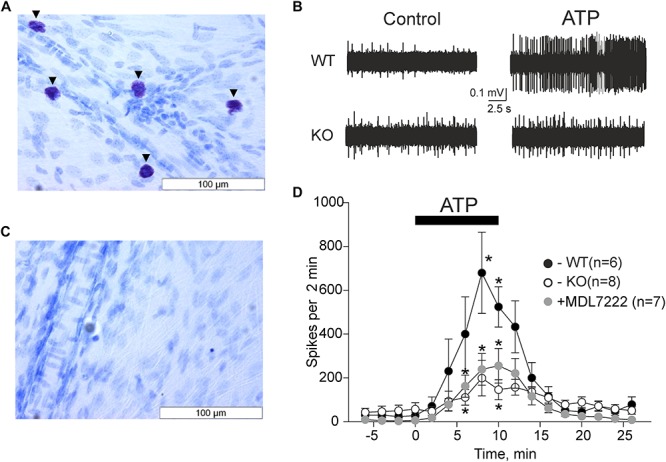
ATP induced meningeal firing was significantly reduced in KO mice compared to WT mice and in the presence of the 5-HT3 antagonist MDL-7222. Toluidine Blue staining of meningeal tissue in the WT **(A)** and KO **(C)** mice. **(A)** Toluidine Blue-stained mast cells are shown along the middle meningeal artery (arrows) in the dura mater. **(C)** KO mice show no Toluidine Blue stained cells. **(B)** Representative traces of nociceptive firing recorded from trigeminal nerve fibers in WT and KO mice, respectively, before and after application of 100 μM ATP. **(D)** The time-course of nociceptive firing during application of 100 μM ATP in the WT (black circles, *n* = 6), KO (white circles, *n* = 8) mice, and 100 μM ATP in the presence of 10 μM MDL-7222 in WT mice (gray circles, *n* = 7). Each point represents a mean spike frequency for 2 min of recording. Mean ± SEM, nerve activation levels compared to the baseline using the Student’s paired *t*-test, *p < 0.05.

We first verified that the mast cell deficient animals were indeed devoid of mast cells. As demonstrated in Figures 1A,C,

where WT meninges contained a vast amount of mast cells, there were no mast cells in the meninges of C57BL/6J-KitW-v/J mice (KO mice).

The pro-nociceptive action of ATP on trigeminal meningeal nerve fibers was electrophysiologically recorded in WT and KO mice. The baseline frequency of meningeal spikes (measured during 2 min before ATP application) was not significantly different in the two groups (27.7 ± 14.8 spikes in the WT, *n* = 6 versus 57.0 ± 29.9 spikes in KO mice, *n* = 8, *p* = 0.322). The application of ATP (100 μM) via rapid perfusion produced a pronounced firing in nerve fibers in both groups of mice (Figure 1B). In WT mice, the frequency of nociceptive spikes after application of ATP increased from the resting value of 27.7 spikes to 400.2 ± 169.1 spikes 6 min after ATP application (*p* = 0.105 as compared to baseline activity, *n* = 6) and to 679.2 ± 185.1 spikes 8 min after ATP application (*p* = 0.024, *n* = 6). In sharp contrast, in KO animals, ATP increased spiking activity from the resting value of 57.0 spikes only to 111 ± 35.5 spikes (*p* = 0.034, *n* = 8) by 6 min and to 199 ± 81.2 spikes (*p* = 0.057, *n* = 8) by 8 min. The detailed time-course of ATP action in WT and KO mice is shown in Figure 1D. Comparative analysis indicated that during the maximal effect (6–8 min of ATP action) the spike frequency in KO mice was significantly lower (*p* = 0.02) compared to the WT mice (Figure 1D).

### MDL-7222 Inhibits ATP Mediated Nociceptive Firing

We recently showed that ATP efficiently promoted the degranulation of meningeal mast cells (Nurkhametova et al., 2019), a process which is associated with the release of multiple active mediators including serotonin. Endogenous serotonin derived from dural mast cells is a likely candidate to excite nerve fibers as it strongly promotes firing of rat meningeal afferents mainly via neuronal ligand-gated 5-HT3 receptors (Kilinc et al., 2017). Therefore, we next investigated the hypothesis that the part of the pro-nociceptive effect of ATP was mediated by endogenous serotonin via 5-HT3 receptors. To this end, we performed experiments where ATP was applied together with the 5-HT3 receptor antagonist MDL-7222. In the presence of this 5-HT3 blocker, ATP (100 μM) was still able to increase the frequency of meningeal spikes from 6.4 ± 2.8 spikes to 160.3 ± 49.9 (*p* = 0.027, *n* = 7) by 6 min, and to 235.9 ± 71 spikes (*p* = 0.023, *n* = 7) by 8 min. However, this effect was significantly (*p* = 0.035) weaker than the peak frequency induced by ATP alone (679.2 ± 185.1 spikes by 8 min, *p* = 0.024, Figure 1D).

### Serotonin Induces Nociceptive Firing via 5-HT3 Receptors

In order to confirm that low concentrations of serotonin close to physiological levels of this monoamine (Nagata et al., 2006; Ćulafic et al., 2007) are active in mice, we applied this monoamine to mouse meninges.

**FIGURE 2:**
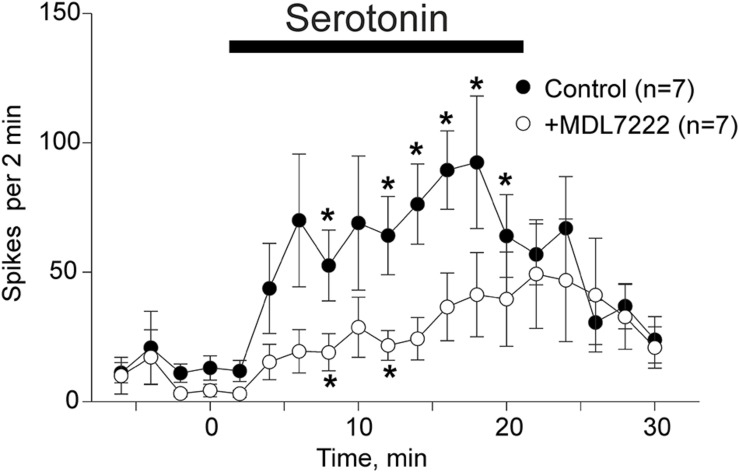
Serotonin induced nociceptive firing and the inhibitory effect of 5-HT3 antagonist MDL-7222 on serotonin-induced activation of meningeal afferents. The time-course of nociceptive firing before and after application of 2 μM serotonin (black circles) and 2 μM serotonin in the presence of 10 μM MDL-7222 (white circles; *n* = 7 in both groups). Each point represents the mean spike frequency of a 2 min recording period. Mean ± SEM, Student’s paired *t*-test, *p < 0.05.

Application of 2 μM of serotonin increased spiking activity of trigeminal nerves in WT mice from 13 ± 4.7 spikes to 89.4 ± 15.1 spikes by 16 min (*p* = 0.002 as compared to baseline activity) and then to 92.4 ± 25.6 spikes by 18 min (*p* = 0.015) after serotonin application (*n* = 7, Figure 2). This excitatory action of serotonin was largely prevented in the presence of the 5-HT3 receptor antagonist MDL-7222 (10 μM) down to 36.6 ± 13.1 spikes by 16 min (*p* = 0.057, *n* = 7, Figure 2) and 41.3 ± 16.3 spikes by 18 min after serotonin applied together with MDL-7222 (*p* = 0.084, *n* = 7, Figure 2).

Comparison of the spike frequency in the period of maximal serotonin-induced activity (14–18 min) showed that the number of spikes was significantly weaker when this agonist was applied together with MDL-7222 (*p* = 0.038, *n* = 7).

### Spectral Analysis of the Pro-nociceptive Effect of ATP

To compare the functional sequences of ATP induced signaling in the presence and absence of mast cells, we performed spectral analysis of firing activity in the meningeal nerves, which normally sends this information to the second order brainstem neurons (Andreou et al., 2015).

**FIGURE 3:**
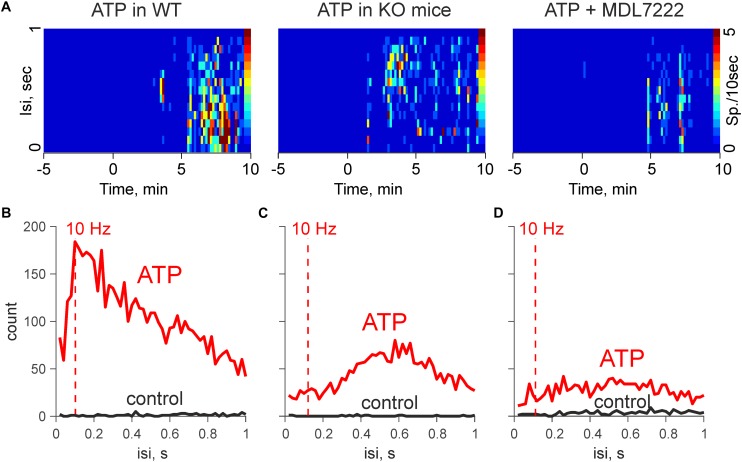
ATP induced high-frequency discharges are reduced in KO mice and in the presence of MDL-7222. **(A)** Heat maps during application of 100 μM ATP in WT (control and in the presence of MDL-7222) and in KO mice. **(B)** Mean interspike intervals during application of 100 μM ATP in WT mice (*n* = 6). **(C)** Mean interspike intervals in control (black) and after application of 100 μM ATP in KO (*n* = 8). **(D)** Mean interspike intervals during application of 100 μM ATP in presence of MDL-7222 in WT (*n* = 7).

Figures 3A,B show that the pro-nociceptive effect of ATP in WT mice was characterized by high-frequency discharges. Notably, the spectral analysis revealed that in the WT mice the activity peaked at 10 Hz, which is sufficient for the temporal summation of excitatory signals at the level of secondary nociceptive neurons (Zakharov et al., 2015). In contrast, in KO mice, spectral analysis indicated a prevailing activity at 0.6 Hz (Figure 3C). Similar results were obtained also in the presence of MDL-7222 (Figure 3D). Thus, in the absence of mast cells, and when the action of serotonin was blocked, ATP-induced high frequency events were significantly reduced.

## Discussion

Here, we demonstrate for the first time the potent excitatory action of extracellular ATP on nociceptive firing of mouse meningeal afferents implicated in generation of migraine pain and the key role of mast cells in this phenomenon.

Despite the high prevalence of migraine, the mechanisms of pain generation in this common disorder have not been fully discovered. The trigeminovascular system of the meninges comprising trigeminal nerve fibers densely innervating dura mater blood vessels, is a well-recognized origin site of migraine pain (Messlinger, 2009; Olesen et al., 2009; Noseda and Burstein, 2013; Pietrobon and Moskowitz, 2013; Zakharov et al., 2015).

Recent evidence also suggests an important role for meningeal mast cells in triggering migraine pain. Thus, mast cells are densely present in meningeal tissues, located adjacent to both nerves and vessels (Theoharides et al., 1995, 2005; Levy et al., 2007). The contact between mast cells and nerve endings forms a neuro-immune synapse where active substances released by mast cells can activate neighboring nociceptive fibers and compounds released from active fibers, in turn, can degranulate mast cells (Dimitriadou et al., 1991). There is a long list of active substances, which can take part in the crosstalk between neurons and mast cells. Thus, degranulation of mast cells leads to release of multiple pro-inflammatory substances including enzymes, neurotrophic factors, pro-inflammatory cytokines, histamine and serotonin (Wernersson and Pejler, 2014; Conti et al., 2019). Degranulation of dural mast cells can strongly activate meningeal nerve fibers (Levy et al., 2007; Kilinc et al., 2017). Interestingly, we found that histamine is weak in excitation of meningeal nerve terminals (Kilinc et al., 2017, see also Schwenger et al., 2007). In contrast, serotonin is a powerful inducer of nociceptive firing in meningeal afferents, operating via ligand-gated 5-HT3 receptors (Kilinc et al., 2017).

One of the endogenous substances, which can activate meningeal afferents, is extracellular ATP, a powerful pro-nociceptive and pro-inflammatory agent (Giniatullin and Nistri, 2013; Burnstock et al., 2014). The purinergic hypothesis of migraine, suggesting an important role of ATP in migraine pathophysiology, was first proposed by Burnstock (1981). We previously showed in rats, that ATP induced nociceptive firing in trigeminal nerves, through ATP-gated P2X3 receptors (Yegutkin et al., 2016; Zakharov et al., 2016). The other study showed that dural topical application of ATP activated more than half of A-delta and C-fibers (Zhao and Levy, 2015). In the current study, we also found that ATP produced a huge (24.6-fold) activation of meningeal trigeminal nerve fibers in mice.

Besides this direct excitatory action on nerve terminals, extracellular ATP is also known as a substance triggering mast cell degranulation (Wareham and Seward, 2016; Nurkhametova et al., 2019). Here, we tested the hypothesis that this concomitant action of ATP contributes to activation of trigeminal fibers via degranulation of dural mast cells and the release of additional excitatory agents, such as serotonin. To test this hypothesis, we used C57BL/6J-KitW-v/J mice deficient in mast cells and found that mast cell deficient mice were significantly less sensitive to the excitatory action of extracellular ATP suggesting that mast cells provided an additional component for the pro-nociceptive action.

As serotonin is a well-known mast cell mediator stored in granules and easily released upon activation (Wernersson and Pejler, 2014), we tested its action on mouse trigeminal afferents. We found that concentrations as low as 2 μM of this biogenic amine are able to excite nerve terminals similar to ATP. Notably, like in rats (Kilinc et al., 2017), this effect of serotonin was antagonized by the specific 5-HT3 antagonist MDL-7222 demonstrating the role of the ligand-gated 5-HT3 receptor as a main target of serotonin.

Moreover, when testing the action of ATP in WT mice, ATP-induced firing was also reduced in the presence of MDL-7222 suggesting that the action of ATP is partially mediated by 5-HT3 receptors. It is worth noting that serotonin can promote release of the migraine mediator CGRP (Kilinc et al., 2017) and contributes to meningeal neuroinflammation (Buzzi and Moskowitz, 2005) which can be a reason for long-lasting pain in migraine. Thus, serotonin can be considered as the endogenous amplifier of purinergic nociception in meninges. On the other hand, at the level of ‘postsynaptic’ neuronal membrane, there could be the inhibitory interactions between 5-HT3 and P2X channels (Barajas-López et al., 2002), which are most significant at high agonist concentrations. This negative mechanism can limit an excessive excitation of afferents when the high level of ATP and serotonin are co-released. ATP-induced firing discharges around 10 Hz detected in the WT and missing in KO mice and in the presence of MDL-7222 may be important for the nociceptive traffic amplification at the level of second order neurons via temporal summation of input signals in the excitatory synapses in the brainstem (Zakharov et al., 2015).

In summary, we report that extracellular ATP, a powerful pro-nociceptive agent, which can be released during a migraine attack (Karatas et al., 2013), stimulates nociceptive firing in trigeminal afferents via a dual mechanism, including degranulation of resident mast cells and by the direct excitatory action on nerve terminals. ATP can be released from multiple cellular sources including astrocytes, neurons, platelets, and endothelial cells, primarily via exocytosis and/or pannexin/connexin hemichannels (Pankratov et al., 2006; Pangrsic et al., 2007; Lohman et al., 2012). Notably, ATP release could be enhanced in migraine-associated conditions such as shear stress and hypo-osmotic cell swelling (Wei et al., 2011; Burnstock and Knight, 2017) and local inflammation (Dosch et al., 2018). We suggest that ATP-driven mechanisms contribute both to excitation and to meningeal neuroinflammation in the local neuro-immune unit formed by dural mast cells and trigeminal afferent fibers.

## Data Availability

The datasets for this manuscript are not publicly available because the raw data supporting the conclusions of this manuscript will be made available by the authors, without undue reservation, to any qualified researcher. Requests to access the datasets should be directed to Rashid.Giniatullin@uef.fi.

## Author Contributions

KK, VG, and OG contributed to data collection, analysis, interpretation, and writing the manuscript. RaisaG contributed to data collection and analysis. DN contributed to writing and editing the manuscript. OM and PL provided the KO mouse line and contributed to writing the manuscript. GS contributed to the study design and supervision of the study. TM and RashidG contributed to the study design and supervision, writing the manuscript, and the final editing. All authors approved the final version of the manuscript.

## Conflict of Interest Statement

The authors declare that the research was conducted in the absence of any commercial or financial relationships that could be construed as a potential conflict of interest.
